# Human Tissue-Resident Memory T Cells in the Maternal–Fetal Interface. Lost Soldiers or Special Forces?

**DOI:** 10.3390/cells9122699

**Published:** 2020-12-16

**Authors:** Caitlin S. DeJong, Nicholas J. Maurice, Stephen A. McCartney, Martin Prlic

**Affiliations:** 1 Vaccine and Infectious Disease Division, Fred Hutchinson Cancer Research Center, Seattle, WA 98109, USA; cdejong@fredhutch.org (C.S.D.); nmaurice@fredhutch.org (N.J.M.); 2 Molecular and Cellular Biology Graduate Program, University of Washington, Seattle, WA 98195, USA; 3 Division of Maternal Fetal Medicine, Department of Obstetrics and Gynecology, University of Washington, Seattle, WA 98195, USA; smccart@uw.edu; 4 Department of Immunology, University of Washington, Seattle, WA 98195, USA

**Keywords:** tissue-resident memory T cells, placenta, maternal–fetal interface, MHC class I/II

## Abstract

The immune system plays a critical role during pregnancy, but the specific mechanisms and immune cell function needed to support pregnancy remain incompletely understood. Despite decades of research efforts, it is still unclear how the immune system maintains tolerance of fetal-derived tissues, which include most cells of the placenta and of course the fetus itself, without forfeiting the ability to protect against harmful infections. T cells recognize antigen in the context of major histocompatibility complex (MHC) encoded proteins, but classical MHC class I and II expression are diminished in fetal-derived cells. Can T cells present at the maternal–fetal interface (MFI) protect these cells from infection? Here we review what is known in regard to tissue-resident memory T (Trm) cells at the MFI. We mainly focus on how Trm cells can contribute to protection in the context of the unique features of the MFI, such as limited MHC expression as well as the temporary nature of the MFI, that are not found in other tissues.

## 1. Human Pregnancy and the Maternal–Fetal Interface

Human pregnancy has a gestational period of 40 weeks, and the role of the maternal immune system continually adapts as the pregnancy progresses. Overall, the immune system appears to play multiple critical roles such as facilitating the fetal-derived placenta to invade and attach to the uterus [1], protecting the fetus from pathogenic threats [2,3], and maintaining tolerance against fetal antigens that are not shared with the mother [1].

**Figure 1:**
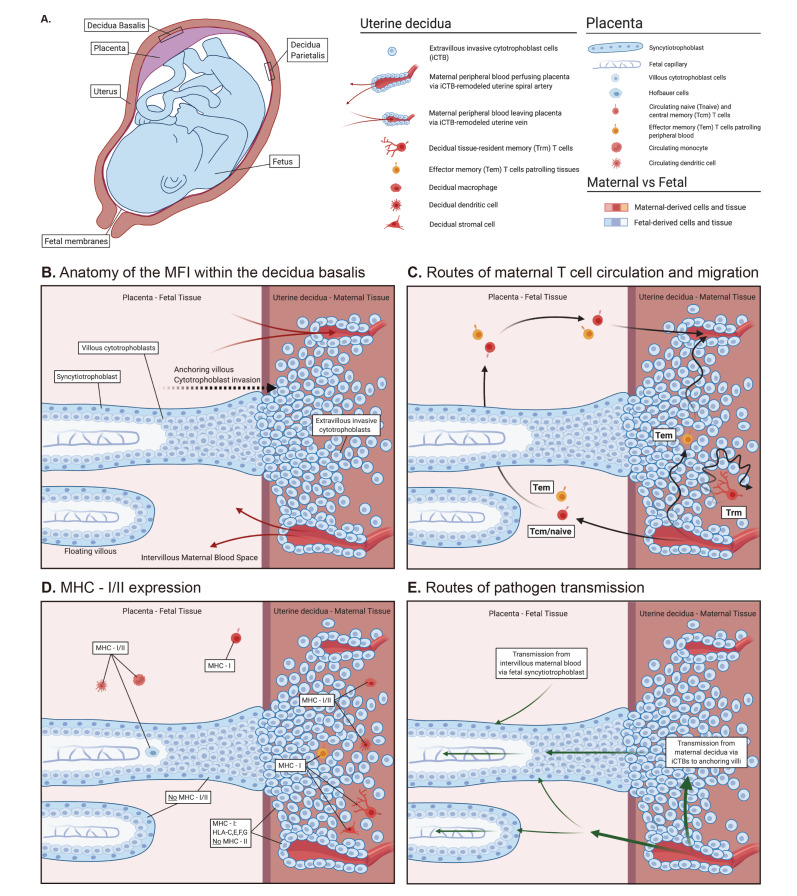
Gross and cellular anatomy of the maternal–fetal interface. (**A**) Gross anatomy of a fetus in utero. Placenta is colored purple to illustrate the maternal uterine (rose) and fetal (blue) tissues intermingling. The region where the placenta anchors to the uterus becomes the decidua basalis; where the fetal membranes contact the remaining surface of the uterus is the decidua parietalis. (**B**) Basic cellular anatomy of the maternal–fetal interface illustrating the fetal cells of the placenta (blue) invading the uterus (rose) and the maternal peripheral blood (light rose) bathing the placental villi. (**C**) Different maternal T cell populations patrolling complementary regions for antigen; intervillous blood space versus decidua. Black arrows indicate routes of surveillance. (**D**) Varied MHC-I and -II expression by maternal and fetal cells. (**E**) Possible routes of pathogen transmission (green arrows) from mother to fetus. Figure created with BioRender.com.

The maternal–fetal interface (MFI) forms upon implantation of the fetal-derived placenta into the maternal endometrium. During implantation, the maternal endometrium undergoes the process of decidualization, which results in remodeling of the maternal spiral arteries, differentiation of maternal stromal cells from fibroblasts to secretory cells, and influx and alterations of immune cells [4]. This specialized endometrial tissue is known as the decidua, which exists only during pregnancy. The decidua consists of two distinct compartments: the decidua basalis, located at the site of placentation which is deeply invaded by fetal cells, and the decidua parietalis, consisting of the remainder of the decidua, which is in contact with the fetal membranes but not deeply invaded by fetal cells [5] (Figure 1A). Significant immune changes occur at the MFI during decidualization. The nonpregnant endometrium consists of 10–20% CD45^+^ leukocytes, predominantly T cells (6–60%) and NK cells (25–85%), which vary in frequency throughout the menstrual cycle [6,7,8,9,10]. The composition of this immune cell population significantly changes during pregnancy. During the first trimester, the decidua consists of 40% CD45^+^ leukocytes, predominantly NK cells (70%), macrophages (20%), and T cells (10–20%), and 40–80% T cells by the end of gestation [9,11,12,13,14,15,16]. Prior to the definition of Trm cells, early work profiling the decidua mononuclear cell population from healthy first-trimester human pregnancies [17,18] identified TCRαβ^+^ cell populations throughout decidual tissue, within the stroma adjacent to endometrial gland and endothelium, as well as intraepithelially [17].

The placental structure at the MFI consists of chorionic villi including both anchoring villi, made up of extravillous trophoblasts which invade deep within the decidua, eventually replacing the maternal endothelium and remodeling the spiral arteries, and floating villi, which float freely within the intervillous space (Figure 1B) [19,20]. The chorionic villi consist of a single outer layer of a fused multinucleated syncytiotrophoblast with underlying cytotrophoblasts that support fetal capillaries. While the intervillous space is supplied with maternal blood, facilitating the transit of gases, nutrients, and wastes across maternal and fetal circulation, the syncytiotrophoblast prevents maternal and fetal blood from directly mixing; the syncytiotrophoblast is formed from underlying trophoblasts fusing into a large multinucleated barrier (Figure 1B).

Maternal T cells are present throughout these substantial changes in tissue composition/function that occur during placentation. We will first provide a general overview of the different T cell subsets and their associated functional properties followed by a closer look at how the T cell compartment, particularly tissue-resident T cells, in the MFI changes during pregnancy.

## 2. Introduction to Memory T Cell Subsets Circulating in Human Blood and Tissues

Before a CD4 or CD8 T cell can contribute to pathogen clearance, they must first become activated, which requires three distinct signals: a naïve T cell, expressing a unique T cell receptor (TCR) to a cognate antigen, receives a T cell receptor signal (Signal 1) in the context of a costimulatory signal (Signal 2) and inflammation (Signal 3) [21]. This priming event occurs in the lymph nodes and requires a professional antigen-presenting cell (APC), such as a dendritic cell, to present antigen in the context of MHC class I or II to naïve CD8 and CD4 T cells, respectively [22]. Of note, these T cell–APC interactions are spatially orchestrated and occur in distinct regions of the lymph nodes [23,24]. Successful T cell priming leads to the acquisition of effector function, such as secretion of interferon-γ (IFNγ), and extensive T cell proliferation [25]. CD8 T cells are particularly efficient at undergoing rapid and robust clonal expansion, and a single CD8 T cell can give rise to >10^4^ antigen-specific progeny [26]. 

The magnitude of this clonal expansion depends on the inflammatory environment as well as the affinity for and availability of antigen [27]. After the peak of this expansion (typically around 1 week after infection in a mouse model of acute infection) follows the contraction phase, in which ~90% of the effector T cells undergo programmed cell death in a Bim-dependent manner [28]. The remaining 10% form a long-lived memory population. This memory population can be further subdivided into central memory, effector memory, and tissue-resident memory based on the respective migratory and functional characteristics [29]. Each of these memory populations is responsible for patrolling different tissue regions within the body in a complementary manner to achieve long-lived immune protection [29]. Of note, all of these mechanistic studies were done using the mouse model system. While we highlight some mouse studies for the general T cell overview, we will exclusively focus on human studies for discussing T cells in the MFI.

A set of biomarkers is used to extrapolate functional and migratory characteristics and serves as the basis for defining each subset: in humans, the CD45 isoforms CD45RA and CD45RO, respectively, serve as indicators of antigen-naïve or memory T cells. CCR7 and CD62L expression are required for T cells to access the lymph nodes via high endothelial venules (HEV) and are used to distinguish central memory (CCR7^+^ CD62L^+^) from effector memory (CCR7^−^ CD62L^−^) T cells in the blood [30]. When examining T cells in nonlymphoid tissues, two biomarkers, CD69 and CD103, typically serve to identify tissue-resident T cells and distinguish them from effector memory T cells and have been used in mouse model studies and human studies [29,31]. Of note, CD69 is also a biomarker for recent T cell activation (TCR- or cytokine-mediated [32]) and thus its expression should be interpreted carefully and in conjunction with other markers or transcriptomic signatures. Below, we briefly summarize the defining biomarkers and functional hallmarks of naïve T cells and each memory T cell subset, which will provide the framework for understanding their potential roles in the MFI.

Naïve T cells: Both naïve CD4 and CD8 T cells express the CD45 isoform CD45RA, as well as CCR7 and CD62L, which are necessary for entering the lymph nodes via the HEVs [30,33]. Of note, priming of naïve T cells occurs in either the lymph nodes or the white pulp of the spleen. Although naïve T cells are typically not located in nonlymphoid tissues, they are the most abundant T cell subset in the blood of most adolescents to middle-aged adults and as such are still present in tissue preparations and biopsies (Figure 1C).

Central memory T (Tcm) cells: Tcm cells are CD45RO^+^ and express CCR7 and CD62L. Thus, Tcm cells have a migration pattern similar to naïve T cells and patrol blood, lymph, and secondary lymphoid tissues [30] (Figure 1C). Tcm cells are typically associated with the ability to rapidly proliferate again upon antigen encounter with a somewhat less pronounced role of secretion of effector molecules such as IFNγ and granzyme B [30,34]. 

Effector memory T (Tem) cells: Tem cells are CD45RO^+^ and are CCR7^−^ and CD62L^−^. Tem cells cannot access lymph nodes via HEVs and instead circulate between blood and nonlymphoid tissues [30]. Tem cells are thought to patrol nonlymphoid tissues to surveil for reinfection (Figure 1C). Tem cells are associated with rapidly exerting effector function after reactivation such as producing high levels of IFNγ [30,34]. 

Tissue-resident memory T (Trm) cells: Trm cells resemble Tem cells in regard to expression of phenotypic markers, including CD45RO^+^, CCR7^−^ and CD62L^−^, and also functional properties such as rapidly exerting effector function after reactivation [35]. The defining feature of Trm cells is their ability to remain in the tissue for prolonged periods of time without exiting and recirculating, which has been demonstrated in human and mouse studies [31,36,37,38,39] (Figure 1C). Of note, whether Trm cells can leave tissues has not been addressed comprehensively, but recent mouse and humanized mouse studies suggest that this can occur [40,41]. Most Trm data stem from mouse studies, but direct evidence for human Trm cells was also provided in a study that examined T cell abundance in a cohort of cutaneous T cell lymphoma (CTCL) patients that received low dose alemtuzumab (anti-CD52 antibody) treatment [31]. While circulating T cells were depleted by the anti-CD52 antibody treatment, T cells with a Tem phenotype in the skin were not depleted despite CD52 surface expression. This suggests that these cells do not leave the skin as they would be depleted in the periphery and stay in the skin for prolonged periods of time, thus justifying their designation as Trm cells. Of note, this patient cohort did not suffer from infections despite the lack of T cells in the blood, which suggests that the Trm populations in the skin and presumably other barrier tissues could provide protection [31]. This highlights another hallmark feature of Trm cells: Trm cells are activated in situ and do not require trafficking to draining lymph nodes for activation [37] (Figure 1C). 

Trm cells have been characterized across a range of human tissues including skin, lung, gut, oral mucosa, and the female reproductive tract [35,42,43,44,45]. Mouse model studies have shown that memory T cells can traffic to tissues regardless of the presence of antigen in the tissue [46,47], but also suggest that in situ infection and antigen availability are critical factors for effective Trm retention [48]. This notion is intriguing in the context of the MFI as it raises the questions of when, how, and why Trm cells are recruited to the MFI.

Finally, reliably identifying Trm cells is challenging due to the lack of a biomarker uniquely expressed by Trm cells. In addition to the aforementioned expression of CD69, expression of CD103 (integrin, alpha E) is another biomarker used to extrapolate tissue-residence of T cells. The combined expression of CD69 and CD103 appears to be T cell subset- (more predominantly on CD8^+^ than CD4^+^ Trm cells [35]) and tissue-dependent [49,50]. In addition to using CD69 and CD103 as biomarkers to extrapolate tissue-residence, a transcriptomic signature has been proposed to identify human Trm cells [49]. 

Other memory T cell subsets: A CD45RA^+^ CCR7^−^ memory T cell population is present in human blood with properties similar to Tem cells. These CD45RA^+^ CCR7^−^ T cells have been referred to as Tem re-expressing CD45RA (Temra) cells [51]. Additional memory T cell subsets have been proposed but are omitted here for the sake of brevity. It is also important to consider that biomarker-based (instead of function-based) subsetting may often merely represent a snapshot in the continuum in memory T cell properties rather than a population with distinct functional properties [29].

Finally, the focus of this review is on Trm cells in the MFI, and we would like to refer to recent reviews that provide a more detailed overview of the current Trm literature [52,53].

## 3. The Memory T Cell Compartment Changes during the Course of Human Pregnancy in the Maternal–Fetal Interface 

### 3.1. Tissue Residency: Preimplantation 

Since tissue residency markers have only recently been defined, limited studies have explored endometrial Trm cells preimplantation. T cells, alongside NK cells, are the major immune populations within the endometrium prior to implantation. Within the T cell compartment, a near 1:1 CD4/CD8 T cell ratio was observed in healthy endometrial biopsies at the secretory phase of the menstrual cycle [54,55,56]. This distribution substantially differs from that of blood (in which CD4 T cells are more frequent) and mirrors that of other barrier tissues that are largely populated by Trm cells, including the endocervix, ectocervix, and vagina [45,57,58]. Indeed, endometrial memory T cells (defined by CD45RO positivity) expressed CD69 (and to a lesser extent, CD8 T cells coexpressed CD103) [54] suggesting that Trm cells are present at the window of implantation. However, the expression of biomarkers needs to be interpreted carefully when extrapolating T cell subset distribution. While most Trm cells have a Tem-like phenotype, those with Temra phenotypes can also contribute to tissue residence [58], yet do not express CD45RO, potentially explaining the reported CD69 expression in CD45RO^−^ endometrial T cells [54]. In line with bona fide tissue residence, CD69^+^ memory T cells isolated from the endometrium express the proteins PD-1 [54] or CCR5 [55] and transcripts for CD49a [59], mirroring phenotypic and transcriptional signatures broadly shared by human Trm cells across mucosal and lymphoid tissues [45,49,57]. While CCR5 expression has long been associated with a Th1 phenotype [60], a recent study demonstrated that CCR5^+^ CD4 T cells in healthy and inflamed human barrier tissue are actually potent IL-17 producers [45]. If CCR5^+^ CD4 T cells at the MFI are Th1- or Th17-like is still unknown. Of note, these signatures have overlap with those associated with T cell activation [49], potentially explaining why studies of the maternal–fetal interface have reported the presence of “activated” lymphocytes [18,54,55,61,62,63,64].

Additional studies have sought to indirectly interrogate the immune profile of the nonpregnant endometrium using menstrual blood, which contains a mixture of blood and endometrial tissue [10,65]. Despite an increase in CD4/CD8 T cell ratios (owing to the presence of both blood and endometrial tissues) [10,65], menstrual blood contains cells with suggestive Trm phenotypes. Bulk T cells isolated from menstrual blood display increased CD69 and CD103 expression [10].

### 3.2. Tissue Residency: Postimplantation

Following the extensive tissue remodeling to the endometrium postimplantation, the resulting decidua also harbors Trm cells. In the early stages of pregnancy, in which trophoblast invasion is shallow, a major subset of Tem cells express CD69 (with a lesser fraction of coexpressing CD103 within CD8 T cells) [66]. While CD103 coexpression in CD69^+^ CD8 T cells in the first-trimester decidua is markedly less than what is observed in other mucosal tissues [45,57,58], it suggests that some degree of TGF-β signaling [67] may occur at the maternal–fetal interface [68]. Although these studies detected a large fraction of CD69-expressing T cells (approximately 75% of CD8s and 50% of CD4s) within the decidua, sizable populations of Tcm and naïve T cells were also detected, indicating the presence of (contaminating) blood (Figure 1C) and possibly even underestimated frequencies of Trm cells [66]. Transcriptomic studies of tissues from 6–14 weeks gestation echoed Trm findings, as *SELL* (encodes CD62L) transcripts were lower and *CD69, PDCD1* (encodes PD-1), *ITGA1* (encodes CD49a), and *ITGAE* (encodes CD103) were enriched in decidual T cells [11]. As pregnancy progresses and placental trophoblasts invade deeper into the maternal decidua, Trm cells appear to remain a sizable fraction of the immune infiltrate [61,69,70].

A recent study used mass cytometry to assess the immune landscape in first-, second-, and third-trimester decidua [70]. This study included paired decidua basalis and parietalis along with maternal blood for the third-trimester cohort. It is noteworthy that the authors used a very thorough approach and included PBMC reference samples in their mass spectrometry experiments, which greatly facilitates controlling for batch effects and other technical issues. Across term deliveries, an enrichment of memory T cells (delineated using CD45RO and CD45RA in conjunction) was observed in decidual tissues (both basalis and parietalis), which was greater than that observed in blood or early (first or second) trimester tissues [70]. Although not directly addressed, the data presented in this study further suggest many of these memory T cells are indeed Trm cells, as indicated by CD69 and PD-1 expression [70]. Interestingly, these cells expressed additional activation markers: HLA-DR, ICOS, TIGIT, and CD39 [70]. As pointed out by the authors in their discussion, further studies are required to determine the significance and functional properties of these T cells.

Regulatory T cells (Treg) can also become tissue-resident; Treg cells with a Trm cell transcriptome upregulate CD69 and additional markers commonly associated with activation, including OX40, 4-1BB, PD-1, HLA-DR, and ICOS, varying in a tissue site-dependent manner [71]. Recent descriptions of Treg cells isolated from term decidua highlight the probability of a decidua-resident Treg cell population that expresses the aforementioned activation markers [72]. Of note, the high expression of activation markers is restricted to the placental bed (i.e., decidua basalis), as opposed to other sites within the uterus, potentially signifying site-specific immune changes to facilitate placentation [72]. The interplay of Treg and Trm cells across MFI tissues is of course of great interest—a recent study reports three distinct Treg populations in the MFI that can differentially suppress T cell effector function [73]. However, the precise role of decidua-resident Treg cells remains unclear. Trm cells isolated from term decidua are able to exert functionality (effector cytokine production in CD8 Trm cells and suppression in Treg Trm cells) after in vitro stimulation [64,72]. Further, the functional profile of stimulated decidual Trm cells mirrors that of stimulated Trm cells from other tissues; i.e., they are capable of producing IFNγ, TNFα, perforin, and granzymes [45,57,58]. These data are in line with the notion that Trm cells are meant to protect the MFI from infection, and future studies will need to determine how, when, and where Trm and Treg cells interact in the MFI to control effector responses.

Although one could argue that the presence of Trm cells at the maternal–fetal interface pre- and postimplantation is not unexpected, it still raises questions in regard to the biological purpose of these cells: are CD8 Trm cells in situ mainly protectors of maternal cells, or do they have other functional responsibilities for fetal-derived cells? Do CD4 Trm cells in the MFI resemble their counterparts in other mucosal tissues? How dynamic are these roles given that Trm cells can respond to and presumably integrate a wide range of environmental signals, including contact with invading trophoblasts, changes or exposure to hormones or proinflammatory cytokines throughout gestation [11,59,74]? Finally, as outlined in the next section, the ability of Trm cells to mediate protection differs in the MFI compared to other tissues. 

## 4. T-Cell-Mediated Protection at the Maternal–Fetal Interface

In order to conventionally exert effector functions, CD8 and CD4 T cells must recognize antigens in the context of MHC class I and II, respectively. In humans, the MHC class I molecules are human leukocyte antigens (HLAs) A, B, C, E, F, and G, and the MHC-II molecules include HLA-DR, -DQ, and -DP [75]. Conventional CD8 T cells are restricted to recognizing antigens in the context of HLA-A, -B, and -C, while HLA-E, -F, and -G are nonclassical MHC-I molecules. HLA-A, -B, and -C are expressed by nearly all nucleated cells, which is critical for CD8 T cells to detect the expression of antigens. MHC class II expression is not nearly as ubiquitous and is highly cell-type-dependent. MHC class I and II expression patterns in the MFI are fairly well characterized [5]. The expression patterns of MHC class I and II can be considered in (broadly speaking) three areas where maternal and fetal cells come in direct contact: (1) the fetal syncytiotrophoblast that overlays the placental villi and is in direct contact with maternal peripheral blood that perfuses the placenta, (2) the maternal decidua basalis where fetal extravillous trophoblasts invade to anchor the placenta and remodel spiral arteries, and (3) maternal decidua parietalis where the fetal membranes come in contact with the remaining surface of the uterine wall (outside of the site of placental implantation) (Figure 1A,B,D). 

Although the syncytiotrophoblast can be in contact with circulating T cells, it does not express MHC class I or II molecules, thus preventing T cells from recognizing alloantigens as well as possible infections in a TCR-mediated manner [1,5,13,76]. Similarly, extravillous invasive cytotrophoblasts (iCTBs) do not express MHC class II molecules and therefore do not present antigen to CD4 T cells [1,13,76]. However, iCTBs express some but not all MHC class I molecules: one classical (HLA-C), and three nonclassical (HLA-E, -F, and -G) [13,76,77,78]. Fetus-specific T cells can expand during pregnancy [14,79,80], and may be shaped to some degree by HLA-C mismatch [81], but are not per se associated with pathologic conditions [14,79,80]. Of note, both virus-specific (i.e., fetal-nonspecific) and fetal-specific T cells can be detected in the decidua of women by HLA-A and HLA-B tetramers [14,82]. The presence of virus-specific cells has led to the postulation that these cells may be poised to protect from transplacental infection [82,83]. 

Possible routes of placental infection: There are two routes by which a pathogen can infect the placenta: (1) via the maternal circulation to the fetal syncytiotrophoblast or (2) via ascending infection through the maternal decidua which directly contacts the placental trophoblast and fetal membranes [79,84] (Figure 1E). The majority of bacterial infections in human pregnancy are thought to occur via the later route through ascending infection from the lower genital tract; however, viral infections are thought to occur predominantly through the hematogenous route [85]. The syncytiotrophoblast is the primary barrier against infection by hematogenous pathogens [84]. The syncytiotrophoblast has several mechanisms to resist hematogenous infection, including lack of cellular junctions from cell fusion, restricted expression of common pathogen cell entry receptors, secretion of antiviral factors, and high basal rate of autophagy [86]. The cellular immune response to hematogenous pathogens in the placenta consists primarily of leukocytes from maternal and fetal circulation (Figure 1C,D). Pathogens associated with ascending infection are thought to initially infect the decidual stroma, which activates the decidual immune cells through inflammatory cytokine production [87,88]. If the immune response is inadequate and infection occurs within the decidua basalis, the pathogen can directly infect the cytotrophoblasts of the anchoring villi and then the villous core and fetal circulatory system (Figure 1E). If uncontrolled infection occurs within the decidua parietalis, the proximity of the fetal membranes results in a fetal infection via the amniotic fluid (Figure 1A). 

Given the diverse ways in which a pathogenic assault may reach the developing fetus, the question arises if there are temporal (as well as anatomical) needs in immune monitoring at the MFI. Within the immune system, there are T cell subtypes with propensities for different locations, and differences in subset distribution and activation have been described between decidua basalis and parietalis [89].

## 5. Open Research Questions Relating to Trm Cells in Pregnancy

We currently understand the utility of Trm cells to provide long-lived tissue and antigen-specific immune protection. As such, one could argue that finding these cells in a tissue site (the endometrium) that experiences monthly cellular turnover (menses) in the absence of pregnancy and the invasion of the fetal-derived (semi-allogeneic) placenta which rapidly comes in and out of existence over the course of nine months during pregnancy is unparalleled. Questions surrounding how and why Trm cells take up residency at this uniquely dynamic tissue site remain largely open. Furthermore, in “classical” tissue sites populated by Trm cells, antigen-specific CD8 Trm-mediated killing and protection is achieved by cells presenting antigen via MHC class I molecules (HLA-A, -B, and -C), yet the syncytiotrophoblast does not express MHC class I or II and iCTBs only express HLA-C. Do Trm cells forego fetal protection in favor of tolerance? Furthermore, it is unclear if Trm cells have distinct functions when interacting with maternal cells versus fetal cells at the MFI. Importantly, T cells are not always functionally constrained by TCR-mediated activation. Mouse and human studies have shown that resident and circulating memory CD8 T cells can exert effector function, including IFNγ secretion and NKG2D-mediated cytolysis of NKG2D ligand-expressing target cells in an inflammation-dependent, TCR-independent manner [90,91,92,93]. This process has been termed T cell bystander activation. Indeed, decidual CD8 T cells (possibly Trm cells as evidenced by PD-1 expression) appear capable of bystander activation, upregulating cytotoxic molecules after IL-12 stimulation [64]. While bystander activated T cells (both circulating and resident) are capable of limiting pathogen spread in a mouse model [90], it is unclear if this is a mechanism of protection against pathogens at the MFI. Finally, future studies will need to address if inflammation-driven activation of Trm cells at the MFI could also be relevant for pregnancy-associated pathologies.
